# Scintigraphic and radiological correlative and confirmative features obviating invasive biopsy in Caffey's disease

**DOI:** 10.4103/0972-3919.63595

**Published:** 2010

**Authors:** M Ranadheer, Santhi Bhushan Murari, N Sujith, Jayanthi, Pushpalatha Sudhakar, VVS Prabhakar Rao

**Affiliations:** Departments of Nuclear Medicine and Pediatrics, Nizam's Institute of Medical Sciences (NIMS), Hyderabad, Andhra Pradesh, India

**Keywords:** Caffey's disease, chronic osteomyelitis, 99m Tc MDP skeletal scintigraphy

## Abstract

Caffey's disease is not a common clinical occurrence; it often poses problems in diagnosis due to its close resemblance to osteomyelitis. Initial plain radiographic diagnosis is sometimes fraught with the limitation of not being able to differentiate it from chronic osteomyelitis. Skeletal scintigraphy is sensitive in localizing the disease activity to the radiological features of the affected regions and the characteristic location of the lesions helps make the diagnosis without resorting to biopsy and further workup.

## INTRODUCTION

Infantile cortical hyperostosis, also known as Caffey's disease, is a self-limiting disorder of unclear etiology. The disease affects infants presenting with clinical features suggestive of osteomyelitis with bone changes, soft-tissue swelling, fever, and irritability.[1] The unusual presentation of the clinical features and its close resemblance to the commonly occurring Osteomyelitis often delays the diagnosis. Radiography is the most valuable diagnostic study in infantile cortical hyperostosis. Skeletal scintigraphy with its correlative findings confirms the diagnosis and avoids a further invasive workup. A case report encountered, with diagnostic and therapeutic challenge is discussed herewith.

## CASE REPORT

**Figure 1:**
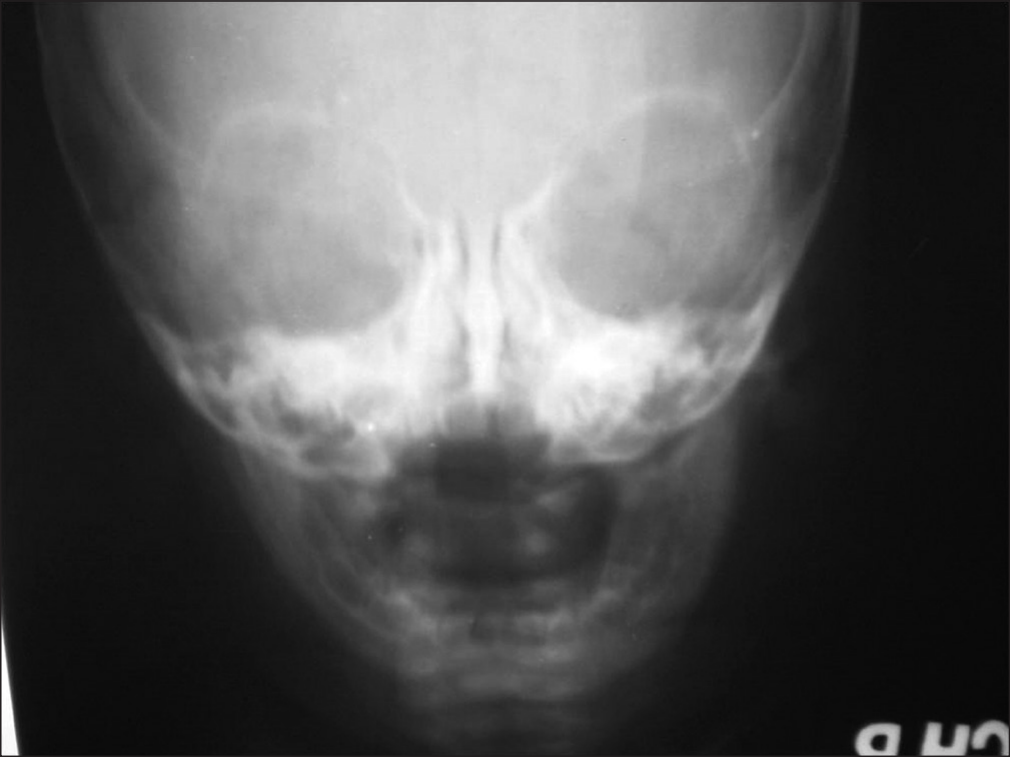
X-ray of the skull showing diffuse thickening and increased density of maxilla and mandibles

A nine-month-old male child presented to the pediatric OPD with irritability, fever, incessant crying, and swelling of face, of 15 days duration. An examination revealed a febrile child, with no localizing signs, except diffuse swelling of the lower jaw associated with tenderness. There were no overlying inflammatory changes in the skin and subcutaneous tissue. The systemic examination was unremarkable. Hematological investigations revealed, Hb 12 gms%, TLC 12600/cc, and ESR 120 mm/h. An X-ray of the skull revealed diffuse thickening of the mandible, with increased density [Figure 1]. The patient was given a course of antibiotics on a clinical diagnosis of osteomyelitis. The child continued to be symptomatic and febrile with no amelioration or worsening of symptoms or signs. The mother noticed a diffuse bony swelling in the upper part of the front of the chest.

**Figure 2:**
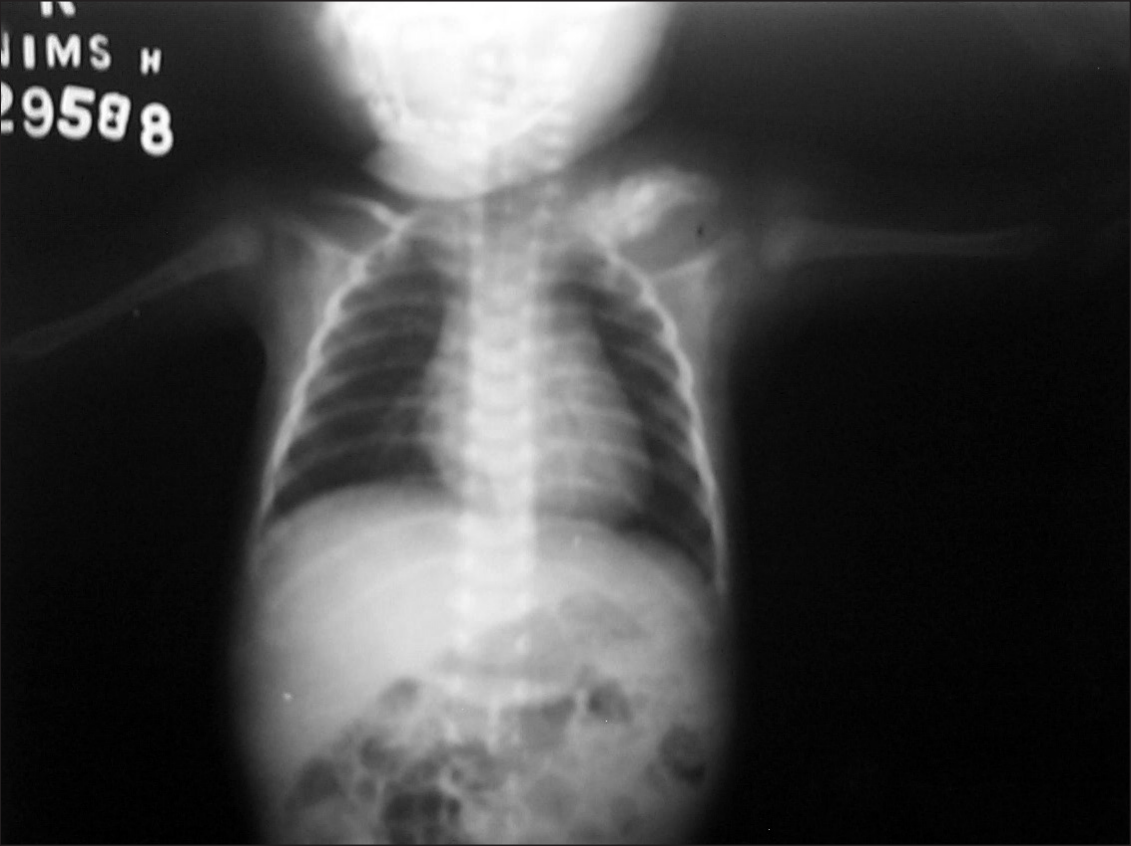
X-ray chest showing bilateral clavicular thickening, sclerosis and overlying soft tissue swelling

Clinically there was diffuse thickening of the left clavicle, which was also tender. An X-ray of the chest revealed unremarkable lung fields and the heart with left clavicular thickening and sclerosis, with associated overlying soft tissue swelling [Figure 2].

**Figure 3:**
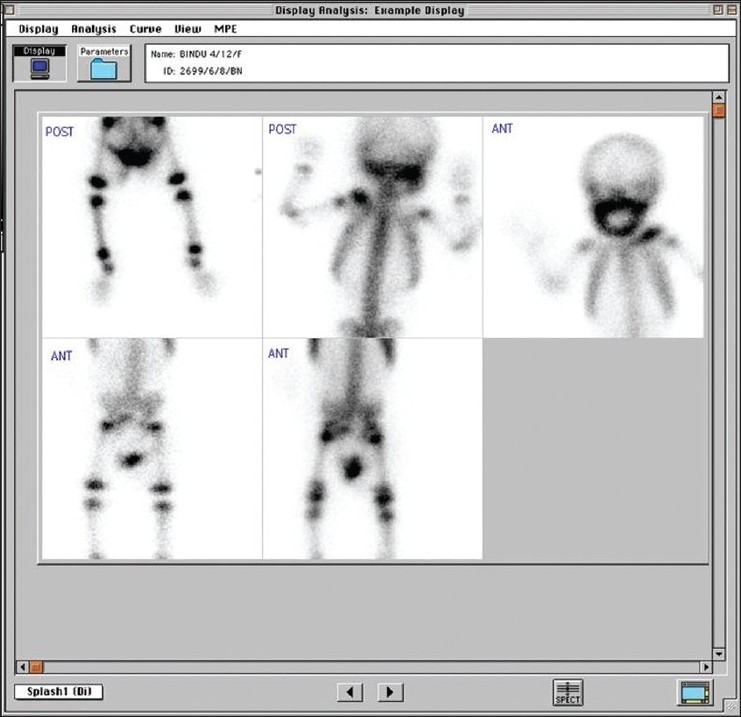
99m Tc MDP skeletal scintigraphy showing diffusely increased uptake in both mandibles, with similar uptake in both clavicles, coinciding with the radiological lesions

In view of the persistence of the symptoms and no change in clinical parameters, the possibility of infantile cortical hyperostosis was suspected and the child was referred for skeletal scintigraphy. 99m Tc MDP Skeletal scintigraphy revealed diffusely increased uptake in both mandibles, with a similar uptake in both clavicles, while the rest of the skeleton showed no active pathological focus [Figure 3]. With the combination of typical radiological findings of diffuse cortical thickening of both mandibles and clavicle and scintigraphic correlation of diffuse avidity in them, and no other incrementing focus of uptake elsewhere, the diagnosis of Caffey's disease was confirmed. The patient's parents were counseled and child put on a short course of steroids due to persisting symptoms. Follow-up after four weeks showed a much improved child, symptomatically.

## DISCUSSION

Infantile cortical hyperostosis, also known as Caffey's disease, is a self-limiting disorder of unclear etiology, which affects infants and causes bone changes, soft-tissue swelling, and irritability.[1] Although the etiology of this condition is not completely understood, familial and sporadic forms[2] appear to exist.

The classic presentation of infantile cortical hyperostosis includes a triad of irritability, swelling, and bone lesions. The swelling appears suddenly, is deep and firm, and may be tender. Fever may occur and babies may refuse to eat, especially if they have mandibular involvement, thus creating an appearance of failure to thrive.[3]

Infantile cortical hyperostosis is often multifocal and asymmetric. The disease has been described in many bones, including the mandible, tibia, ulna, clavicle, scapula, ribs, humerus, femur, fibula, skull, scapula, ilium, and metatarsals.[4] Differential diagnosis includes osteomyelitis, battered baby syndrome, and congenital syphilis. It may recur and rarely cause deformities.[5]

The unusual occurrence of the clinical entity and its close resemblance to the commonly occurring Osteomyelitis often perplexes the clinician to suspect the disease.[6] Radiography is the most valuable diagnostic study for infantile cortical hyperostosis. Radiographs show layers of periosteal new bone formation, with cortical thickening in variable combinations of the long bones, mandible, and clavicle. Scintigraphy, although nonspecific in this entity, is useful for confirming the diagnosis by its correlative avid uptake in the radiologically involved bones, with no other metastatic focus.[7] It also obviates the need for any further workup and invasive biopsies. The classical and correlative radiological and Tc 99M MDP skeletal scintigraphic features make Gallium 67 and leukocyte scintigraphy superfluous and infructuous.[8]

## CONCLUSION

Infantile cortical hyperostosis is an uncommon entity presenting with protean clinical manifestations simulating osteomyelitis and the diagnosis is one of exclusion due to non-improvement with antibiotics for suspected osteomyelitis. The radiological features with diffuse mandibular, clavicular thickening, and scintigraphic features of diffuse non-focal involvement of mandible and clavicles confirm the osteoblastic process, excluding other bony foci. The clinical, radiological, and scintigraphic triad, due to the classical nature, confirms the diagnosis of Caffey's disease precluding the requirement for any further investigations and invasive biopsies. Choice of steroids is empirical to ameliorate pain and the child's suffering.
